# Reversible and Dynamic Tuning Mechanism for Fabry–Pérot Polaritonic Resonators

**DOI:** 10.1002/nap2.70074

**Published:** 2026-04-16

**Authors:** Zhiqiang Shi, Yongqian Zhao, Ying Liao, Rao Fu, Chen Ge, Shuai Feng, Jianing Chen

**Affiliations:** ^1^ School of Science Minzu University of China Beijing China; ^2^ Beijing National Laboratory for Condensed Matter Physics Institute of Physics Chinese Academy of Sciences Beijing China; ^3^ School of Physical Sciences University of Chinese Academy of Sciences Beijing China; ^4^ Suzhou Laboratory Suzhou Jiangsu China; ^5^ Songshan Lake Materials Laboratory Dongguan China

**Keywords:** Fabry–Pérot resonators, near‐field optics, phonon polaritons, polaritons

## Abstract

Polaritons are quasiparticles formed via light‐matter interaction (e.g., with electrons or phonons). They confine light to subwavelength scales, enhance electromagnetic fields in Fabry–Pérot polaritonic resonators, and tune cavity resonance via adjusting external parameters (light frequency or dielectric environment). Thus, we propose tuning Fabry–Pérot polaritonic resonators by modifying the dielectric environment through substrate phase transitions. We perform mid‐infrared nanoimaging of α‐MoO_3_ nanocavities on phase‐change VO_2_ at varying temperatures. At 25°C (VO_2_ insulating phase), adjusting incident light frequency tunes the highly sensitive Fabry–Pérot phononic polaritonic resonator order from 10 to 2; at 90°C (VO_2_ metallic phase), the order tunes from 7 to 2. With incident light fixed at 992 cm^−1^, the α‐MoO_3_ nanocavity/VO_2_ heterojunction undergoes a low‐high‐low temperature cycle, showing a maximum Fabry–Pérot resonance order change of 3, along with reversible tuning ability and delayed recovery. Simulations indicate higher‐order Fabry–Pérot resonant modes can be achieved in thinner and wider α‐MoO_3_ nanocavities. This method provides new means and experimental support for designing tunable Fabry–Pérot resonant devices.

## Graphical abstract

We perform mid‐infrared nanoimaging on an air/α‐MoO_3_ nanocavity/VO_2_ heterojunction and realize dynamic, reversible tuning of phonon polariton resonances in a Fabry–Pérot nanocavity. This tunability arises from dielectric modulation via the thermally driven insulator‐metal transition of VO_2_, enabling nanoscale control of polariton behavior and offering a versatile platform for high‐performance tunable polaritonic devices.

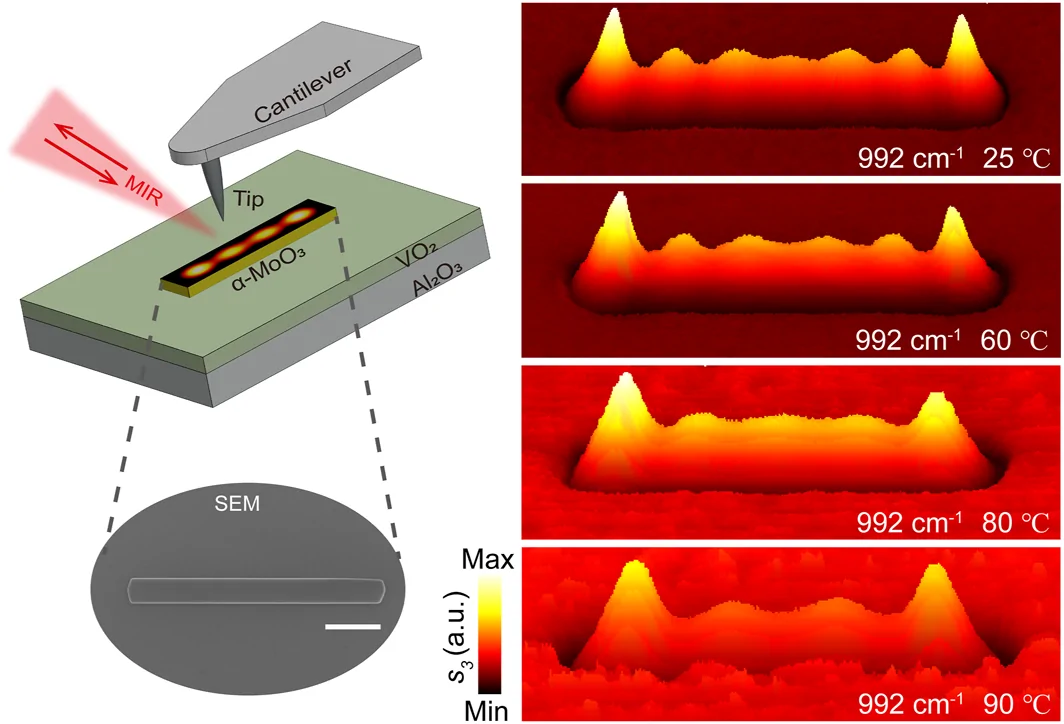

## 1 Introduction

In modern nanophotonics, polaritons have emerged as a focal point of cutting‐edge research due to their ability to confine light beyond the diffraction limit and endow optical fields with matter‐like properties [1, 2, 3]. Moreover, polaritons can significantly enhance the optical field intensity in structures such as Fabry–Pérot (F‐P) resonators [4, 5, 6], providing a powerful platform for manipulating light–matter interactions at deep‐subwavelength scales [7, 8, 9, 10, 11]. The dynamic tunability of polaritons also enables flexible control of F‐P cavity resonance. These features not only overcome the limitations of conventional optics but also open new possibilities for nanophotonic device design [12, 13, 14]. Methods for the dynamic tuning of polaritons have advanced rapidly. Notably, several approaches stand out: driving topological transitions of hybrid polaritons via modulation of the graphene Fermi level [15], controlling polariton dispersion by adjusting the doping level in monolayer graphene [16] and dynamically tuning polaritons in monolayer and twisted bilayer structures through back‐gate voltage to modify the graphene Fermi level [17]. In recent years, polariton F‐P resonators based on materials such as hBN and metallic single‐walled carbon nanotubes have been reported [11, 18, 19, 20]. However, the exploration of external physical tuning methods for dynamically adjusting the characteristics of F‐P resonators has been limited in these studies. Therefore, achieving dynamic tuning of polariton‐based F‐P resonators remains a highly challenging task in this research field.

α‐MoO_3_, a van der Waals material [21, 22, 23, 24], has three Reststrahlen bands in the mid‐infrared region. These bands include Band 1 (545–851 cm^−1^) along the [001] crystal direction (*y*‐axis), Band 2 (820–972 cm^−1^) along the [100] crystal direction (*x*‐axis), and Band 3 (958–1010 cm^−1^) along the [010] crystal direction (*z*‐axis). In the Band 3 region, phonon polaritons (PhPs) can propagate along the [001] and [100] crystal directions [25, 26, 27, 28, 29, 30]. When α‐MoO_3_ is fabricated into a nanocavity, the PhPs resonance of α‐MoO_3_ manifests as F‐P optical resonance modes.

Vanadium dioxide (VO_2_), as a typical and promising temperature‐induced phase‐change material, exhibits a reversible temperature‐induced insulator‐metal transition (IMT), accompanied by abrupt and rapid changes in dielectric properties and optical properties [31, 32, 33, 34]. Therefore, when performing mid‐infrared nanoimaging on α‐MoO_3_ nanocavities spin‐coated onto the surface of VO_2_ films using a scattering‐type scanning near‐field optical microscope (s‐SNOM, Attocube neaspec GmbH) equipped with a quantum cascade laser (QCL, DRS Daylight Solutions), the temperature of the air/α‐MoO_3_ nanocavity/VO_2_ three‐layer heterojunction system was simultaneously regulated. By tuning the incident light frequency *ω* (989.5–998.5 cm^−1^), the F‐P resonance order of the α‐MoO_3_ nanocavity decreased from 10 to 2 at 25°C and from 7 to 2 at 90°C. When ω is fixed at 992 cm^−1^ and the entire α‐MoO_3_ nanocavity/VO_2_ heterojunction system undergoes a complete temperature cycling process, a maximum shift of 3 resonance orders can be observed; during this process, the F‐P cavity resonance order exhibits reversible tuning ability and a delayed recovery phenomenon. Both experiments and simulations confirmed that phase transition control of PhPs F‐P resonance is effective. The mechanism of precisely tuning the F‐P resonant mode of phonon polaritons in α‐MoO_3_ nanocavity through the VO_2_ IMT provides a new strategy for the tuning of polaritonic resonant cavities.

## 2 Basic Optical Characterization of α‐MoO_3_ Nanocavity

Figure 1a shows the configuration diagram of this experiment in which the α‐MoO_3_ nanocavity is transferred onto a 50‐nm thick VO_2_ film grown on a c‐Al_2_O_3_ substrate, and the morphology is given under a scanning electron microscope. We used a s‐SNOM, equipped with a mid‐infrared monochrome continuously adjustable QCL, to perform near‐field optical imaging of a single α‐MoO_3_ nanocavity on VO_2_ under normal pressure. When the atomic force microscopy (AFM) scans the sample below, the probe is irradiated with continuous mid‐infrared monochromatic light, and the scattered optical signal is collected. The incident light is converted by the metallic tip and reflected at both ends of the cavity, and then scattered by the tip again. The scattered signal contains information about PhPs and topography. Figure 1b,c shows polar plots of the Raman spectrum of the α‐MoO_3_ nanocavity from 50 to 1050 cm^−1^ and its polarization Raman peak intensity in *A*_*g*_ (158 cm^−1^) mode. Since the Raman peak in *A*_*g*_ (158 cm^−1^) mode originates from vibrations of transverse rigid chains along crystal directions, its intensity shows significant dependence on the polarization angle [35, 36, 37, 38]. It can be concluded from Figure 1c that peak intensity decreases as the angle increases between 0° and 90°, confirming that the long axis of α‐MoO_3_ nanocavity is collinear with its [001] crystal direction. The real part of the relative permittivity for α‐MoO_3_ depicted in Figure 1d shows three Reststrahlen bands: Band 1 (545–851 cm^−1^), Band 2 (820–972 cm^−1^), and Band 3 (958–1010 cm^−1^) [39]. Among these bands, Band 2 cannot support PhPs propagation along the [001] crystal direction and is therefore not considered here. Both Band 1 and Band 3 support PhPs propagation along [001], whereas PhPs in Band 3 exhibit a substantially higher sensitivity to the illumination frequency [21]. Therefore, the special dispersion behaviors of PhPs in Band 3 area are the basis for precisely tuning the F‐P cavity of α‐MoO_3_ nanocavity PhPs.

**FIGURE 1:**
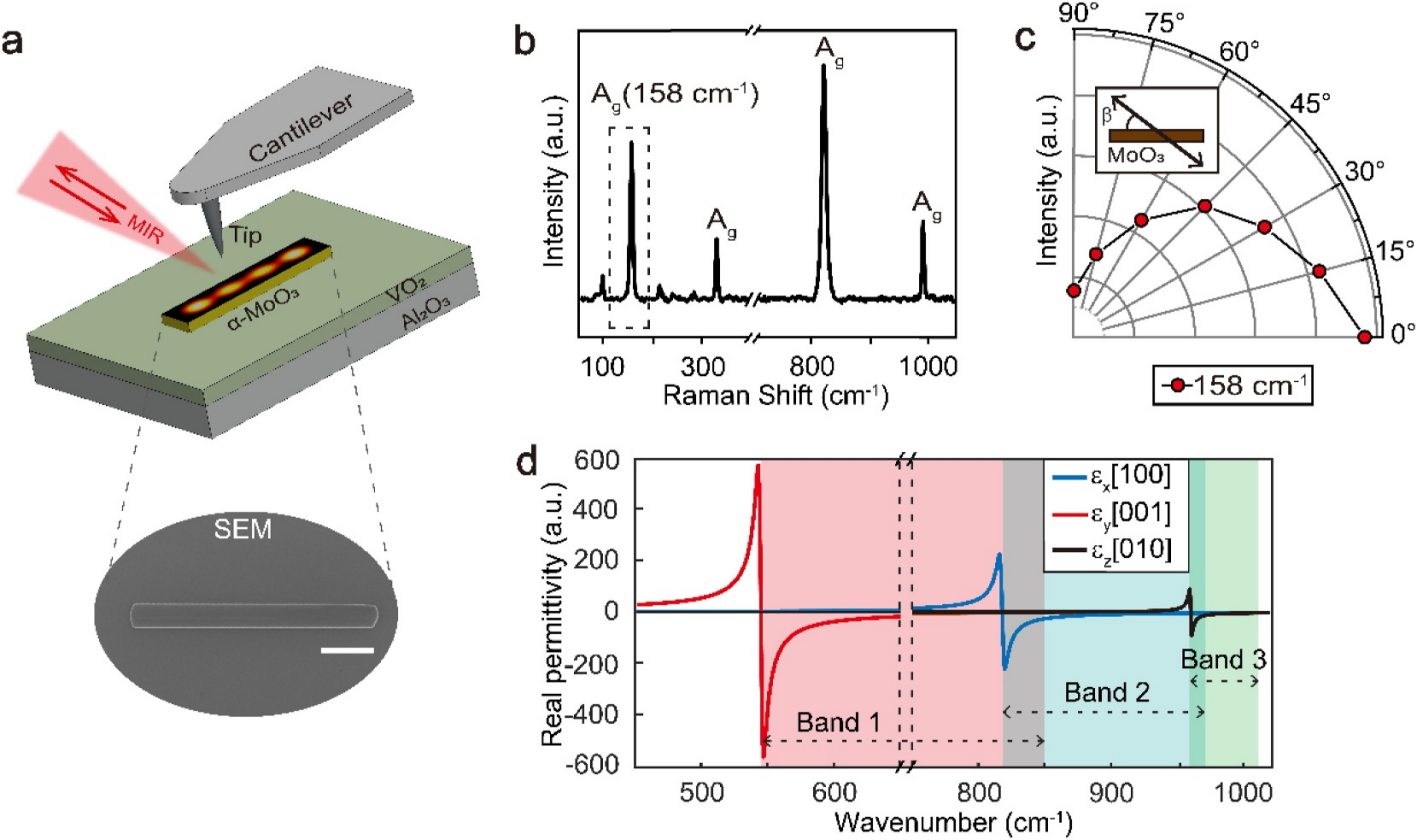
Near‐field optical experimental setup and optical properties of α‐MoO_3_ nanocavity. (a) Schematic diagram of the near‐field optical microscope experimental setup. α‐MoO_3_ nanocavities are transferred to a VO_2_ film grown on an Al_2_O_3_ substrate. The morphology is shown in an image obtained through scanning electron microscopy, with a scale bar of 300 nm. A mid‐infrared laser beam is focused on a metallic tip, and the excited PhPs are reflected at the edge of the cavity, then scattered by the tip, and finally detected by an external detector. (b) Raman spectrum of the α‐MoO_3_ nanocavity. (c) Polar coordinate plot of the intensity of one *A*_*g*_ mode (shown within the dashed box of (b), at 158 cm^−1^) as a function of *β*. The brown rectangular block within the rectangular box represents the top view of the α‐MoO_3_ nanocavity, whereas the bi‐directional arrows indicate the polarization direction of the mid‐infrared laser, with *β* representing the angle between the laser polarization direction and the long axis of α‐MoO_3_ nanocavity. (d) Real part of relative permittivity of α‐MoO_3_ in the range from 450 to 1020 cm^−1^. The red solid line, blue solid line, and black solid line represent the real part of permittivity for α‐MoO_3_ along [001], [100], and [010] crystal directions, respectively.

## 3 Imaging of Non‐Constant Temperature Nanocavity PhPs and Analysis of Their Dispersion

To investigate the F‐P cavity characteristics of α‐MoO_3_ nanocavity at different temperatures, we selected an α‐MoO_3_ nanocavity with dimensions of 1302 nm in length, 160 nm in width, and 40 nm in thickness on a VO_2_ substrate as the research object (Figure 2a,b). It is worth noting that, according to the previous report, even when the VO_2_ substrate is in the metallic phase at 92°C, PhPs in α‐MoO_3_ propagating along the [001] direction still exhibit a propagation trajectory of up to ∼1.5 μm, which further increases to ∼2.5 μm when VO_2_ is in the insulating phase [27]. In this work, the high‐quality α‐MoO_3_ nanocavities used have cavity lengths of 900 and 1302 nm, which are substantially shorter than the propagation trajectory of PhPs on the metallic‐state VO_2_ substrate. Therefore, whether at high or low temperatures, the F‐P resonances along the [001] direction are well satisfied. Consistent with this analysis, well‐defined F‐P resonance modes are indeed observed experimentally. At 25°C (VO_2_ insulating phase), the nanocavity exhibits high‐contrast F‐P cavity interference patterns along the [001] crystal direction (Figure 2c,e). By scanning the ω (from 989.5 to 998.5 cm^−1^), the number of bright spots *j* was observed to range from 9 at 989.5 cm^−1^ to 1 at 998.5 cm^−1^ (more details see Supporting Information S1: Figure S1a,c). At the highest observable F‐P resonance order (i.e., at 989.5 cm^−1^), the electromagnetic confinement capability of the α‐MoO_3_ nanocavity reaches 35.58 ($\lambda_{0}/\lambda_{p}$). When the ω were 992, 993, and 994 cm^−1^, the numbers of bright spots *j* distributed along the [001] crystal direction were 7, 6, and 5, respectively; according to the F‐P cavity resonance order relation (n=j+1), the corresponding *n* were 8, 7, and 6, respectively. The results show that in the mid‐infrared band, only a 1 cm^−1^ tuning of the ω is required to achieve a single‐step change in the resonance order (Δn/Δω=1), highlighting the extremely high sensitivity of this system for controlling the F‐P cavity. The phenomenon that the resonance order decreases with the increase of the ω in the range of 989.5 cm^−1^ to 998.5 cm^−1^ at 25°C and 90°C VO_2_ conditions can be explained by the phase‐matching condition of the F‐P cavity:

(1) $$\begin{array}{c} \frac{2\pi }{\lambda_{p}}L+\Delta \varphi =\pi n \end{array},$$

where L represents the length of the α‐MoO_3_ nanocavity, $\lambda_{p}$ represents the wavelength of PhPs, Δφ (Δφ≈π) is the effective reflection phase by the ends (see Supporting Information S1: Figure S2) [19]. When the temperature is adjusted to 90°C (VO_2_ metallic phase), the F‐P interference pattern in the [001] crystal direction changes significantly: the number of bright spots *j* at 989.5 cm^−1^ decreases from 9 to 6, and the number of bright spots at 996.5 cm^−1^ decreases from 3 to 1; the highest resonance order decreases to 7, as shown in Figure 2d,f (more details see Supporting Information S1: Figure S1b,d). When VO_2_ undergoes the IMT, the real part of its relative permittivity becomes negative. These abrupt dielectric changes induced by the substrate phase transition increase the PhPs wavelength in the α‐MoO_3_ nanocavity, thereby causing a systematic reduction in the F‐P resonance order. (for details, please refer to Supporting Information S1: Figure S6). Moreover, the high loss in the metallic phase (a significant increase in the imaginary part of the VO_2_ relative permittivity) shortens the photon lifetime and selectively eliminates the F‐P higher‐order modes (n= 10, 9, 8) [40, 41, 42].

**FIGURE 2:**
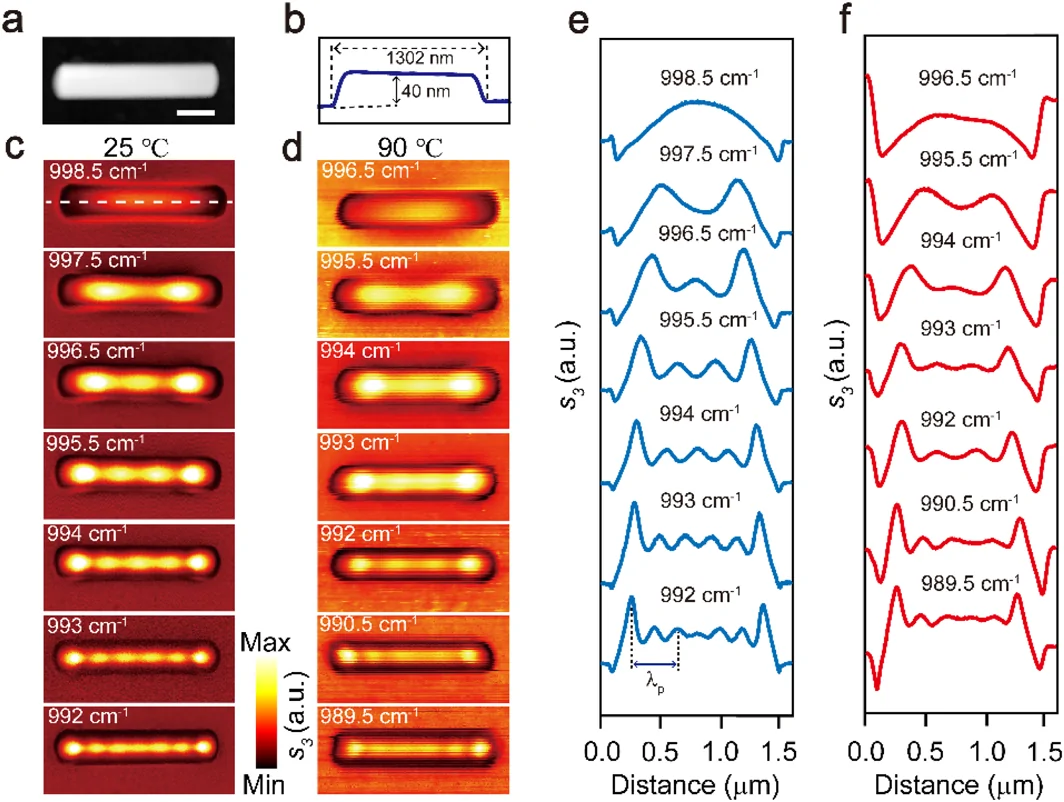
Tuning of incident light in F‐P modes. (a, b) AFM image of a single α‐MoO_3_ nanocavity and its line profile along the center of the nanocavity axis, scale bar of 300 nm. (c, d) Near‐field optical images of a single α‐MoO_3_ nanocavity at 25°C and 90°C, respectively. (e, f) Line profiles along the center of the nanocavity axis corresponding to figures (c) and (d).

We simulated the dispersion relation of PhPs in α‐MoO_3_ nanocavity along the [001] crystal direction to analyze the influence of VO_2_ at 25°C and 90°C on the F‐P cavity resonance. In Figure 3a,b, we extracted the wave vector q ($q=2\pi /\lambda_{p}$) values of PhPs at 25°C and 90°C, respectively, from Figure 2c,d. At the same ω, the q of the PhPs at 90°C is smaller than that at 25°C. However, within the scanning ω range of Band 3 at both 25°C and 90°C, the PhPs follow a trend: as the ω increases, the q of the PhPs decreases, and the resonance order decreases. The experimental phenomena at 25°C and 90°C indicate that the phase transition characteristics of VO_2_, causing changes in the dielectric environment, do not alter the fundamental trend of the PhPs in the α‐MoO_3_ nanocavity within the Band 3 range. Next, we calculated the imaginary part of the complex reflectivity $r_{p}\left(\omega ,q\right)$ along the [001] crystal direction of the α‐MoO_3_ in the air/α‐MoO_3_ nanocavity/VO_2_ three‐layer structures at different temperatures based on the Fresnel formula (see Supporting Information S1: Note S2) and plotted the pseudo‐color map:

(2) $$\begin{array}{c} r_{p}\left(\omega ,q\right)=\frac{r_{a}\;+\;r_{s}e^{i2k_{2}^{b}d}}{1\;+\;{r_{a}r}_{s}e^{i2k_{2}^{b}d}} \end{array},$$

$r_{a}$ and $r_{s}$ represent the reflectivity of the air/α‐MoO_3_ nanocavity interface and the α‐MoO_3_ nanocavity/VO_2_ interface, respectively. $k_{2}^{b}$ denotes the incident light's wave vector along the [001] crystal direction, whereas *d* signifies the thickness of the α‐MoO_3_ nanocavity [27]. Within the range of 960 to 1020 cm^−1^, the dispersion relation of the PhPs along the [001] crystal direction for the air/α‐MoO_3_ nanocavity/VO_2_ system was calculated. As shown in Figure 3a,b, at the same ω, the q of the PhPs at 25°C is larger compared to that at 90°C. This is because when VO_2_ transitions to the metallic phase, its refractive index for light in the mid‐infrared wavelength range decreases, leading to a synchronous reduction in the wave vector [31]. The trend extracted from the experimental data aligns with this observation, and a high correlation is demonstrated between the experimental data and the pseudo‐color map.

**FIGURE 3:**
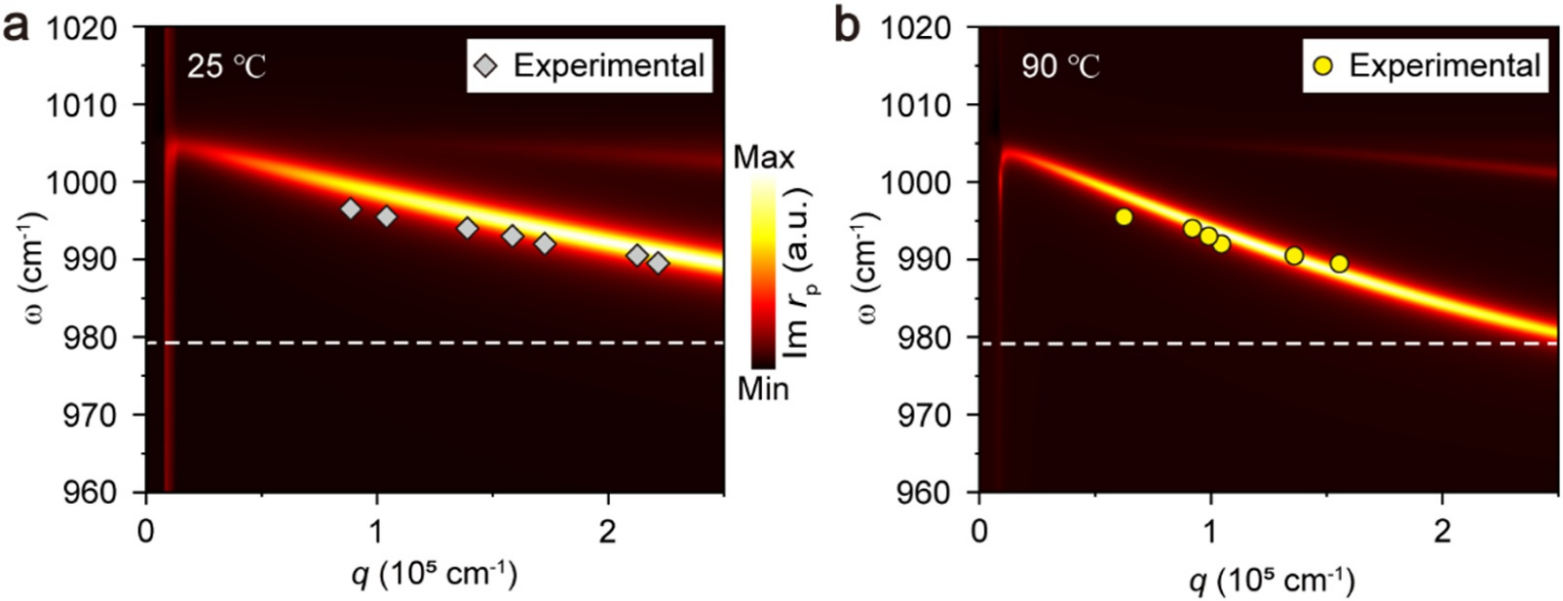
Dispersion relations of α‐MoO_3_ nanocavity PhPs on VO_2_ before and after phase transition. (a) Pseudo‐color map showing the simulated PhPs dispersion of the α‐MoO_3_ nanocavity along the [001] direction at 25°C, where gray diamond blocks represent the PhPs dispersion extracted from Figure 2c. (b) Simulated PhPs dispersion of the α‐MoO_3_ nanocavity along the [001] direction at 90°C, where yellow small circles represent the PhPs dispersion extracted from Figure 2d. The white dashed line is the isoline of the same frequency.

To investigate the detailed tuning mechanisms of the F‐P resonator for PhPs during the IMT process of VO_2_, we maintained the ω at 992 cm^−1^ and performed temperature rise‐fall cycles on the α‐MoO_3_ nanocavity/VO_2_ heterojunction, collecting near‐field images of the PhPs. The temperature range of the cycle was from 25°C to 90°C (more details see Supporting Information S1: Figure S3). During the heating part, the number of bright spots j of the α‐MoO_3_ nanocavity PhPs decreased as the temperature rose: from 7 at 25°C to 4 at 90°C (as shown in Figure 4a). During the cooling part, the number of bright spots j of the α‐MoO_3_ nanocavity PhPs increased as the temperature dropped, recovering from 4 at 90°C to 7 at 25°C (as shown in Figure 4b). Similarly, the F‐P resonance order of the PhPs followed this trend. Next, we extracted the q‐values of the α‐MoO_3_ nanocavity PhPs at each temperature gradient (as shown in Figure 4c) and found that within the range of 30°C–90°C, the q‐values of the PhPs decreased as the system temperature increased; during the cooling process, however, the q‐values recovered to their original values as the system temperature decreased. The temperature‐dependent q curve does not overlap with that of the heating process, showing a certain degree of hysteresis. The temperature at which VO_2_ undergoes IMT is approximately 68°C. At this temperature, its dielectric properties undergo a drastic change [31, 43]. We can observe from Figure 4c that the q‐values of PhPs change most sensitively when approaching this temperature. The temperature sensitivity of PhPs and their hysteresis recovery characteristics during the rise‐fall process provide a new mechanism for tuning F‐P cavities.

**FIGURE 4:**
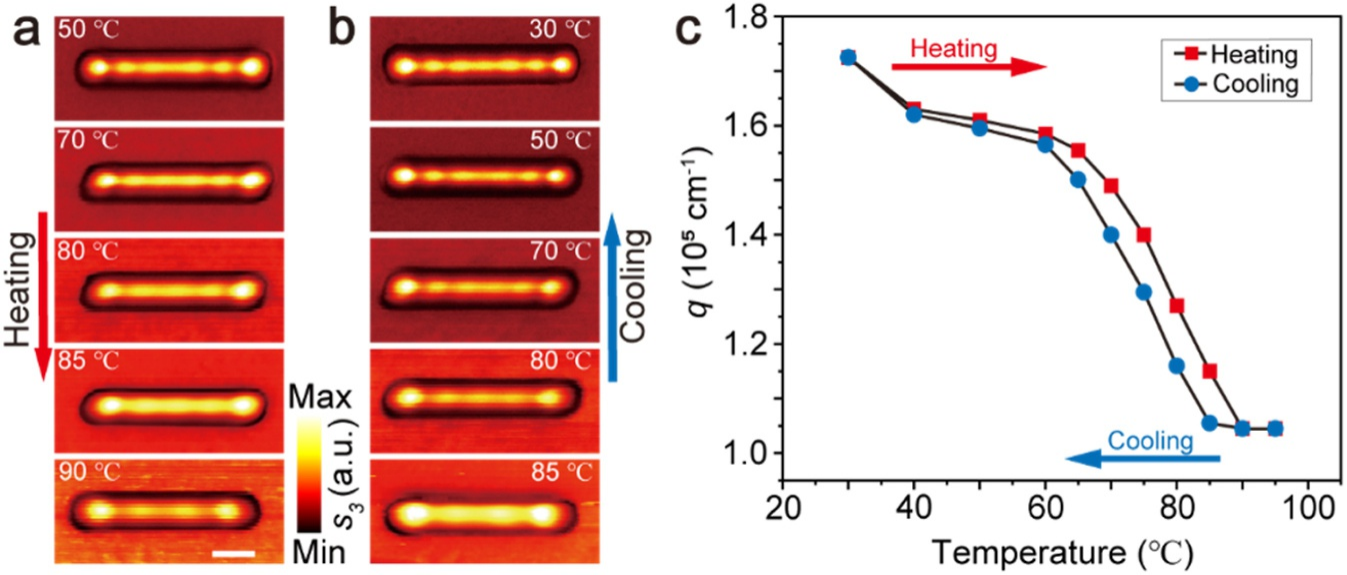
Temperature‐tuned F‐P modes. (a, b) Near‐field optical imaging maps of the α‐MoO_3_ nanocavity/VO_2_ during heating and cooling, with the incident light fixed at 992 cm^−1^. The scale bar is 300 nm. (c) The red and blue dots represent the corresponding PhPs resonance wave vectors extracted from (a) and (b), respectively.

## 4 The Influence of Cavity Size Variation on F‐P Resonance

Given the relatively uniform dimensions and limited variations of high‐quality nanocavities, we investigated the effect of size on the F‐P mode via numerical simulations. Firstly, we changed the width of the α‐MoO_3_ nanocavity while keeping the incident light wavenumber at 992 cm^−1^, the nanocavity thickness fixed at 40 nm, and the length fixed at 1302 nm. We simulated the optical imaging of α‐MoO_3_ nanocavities of different widths before and after the IMT of VO_2_. As shown in Figure 5a,c, under the conditions of VO_2_ at 25°C and 90°C, respectively, it is observed that the resonance order of the F‐P cavity increases with the increase of the width of the α‐MoO_3_ nanocavity, whereas the value of $\lambda_{p}$ decreases. This indicates that the variation in cavity width does indeed have a tuning effect on the F‐P resonance of PhPs (more images are shown in Supporting Information S1: Figure S4). The left and right parts of the red dashed box correspond to the images at 992 cm^−1^ under the same experimental conditions in Supporting Information S1: Figure S1a and Figure 2d, respectively. It can be seen that the simulated near‐field images are highly consistent with the experimental images. This also confirms from the simulation that the IMT of VO_2_ regulates the F‐P resonance order of the PhPs in the α‐MoO_3_ nanocavity.

**FIGURE 5:**
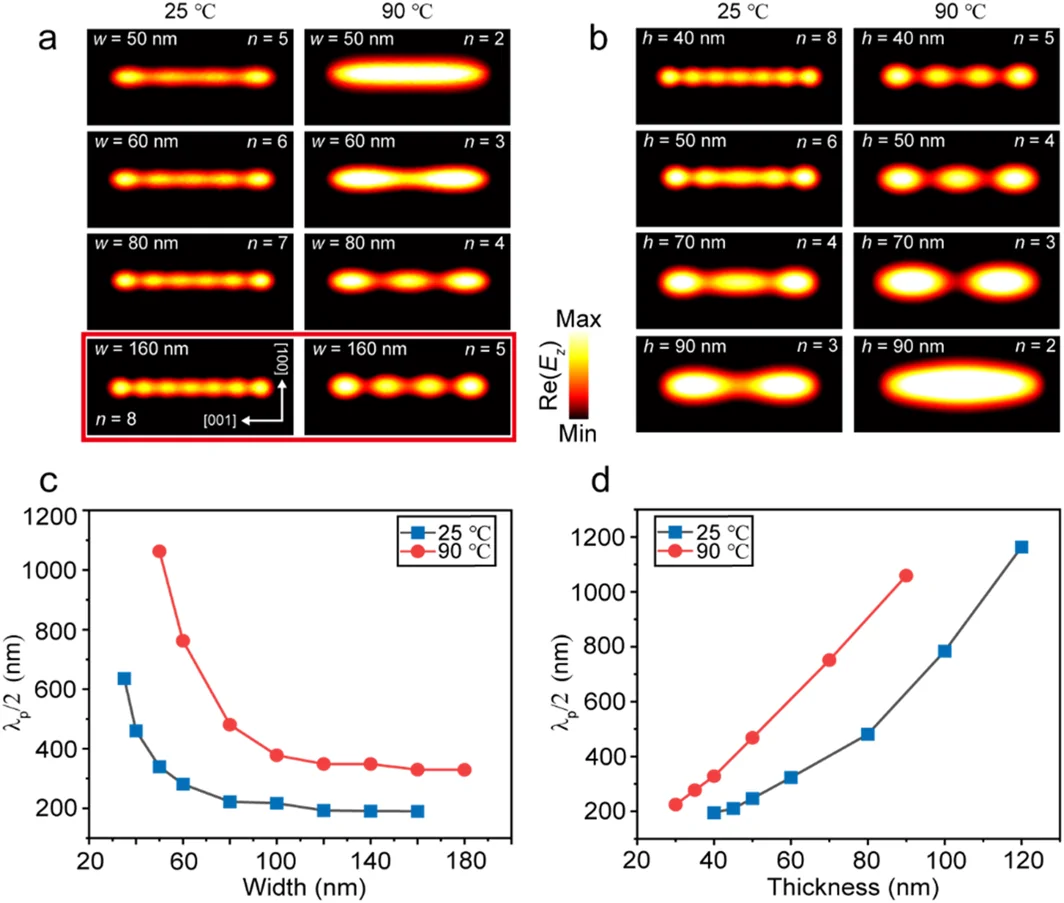
The F‐P cavity modes are tuned through the geometric structure before and after the VO_2_ phase transition. (a) Optical distribution maps of simulated α‐MoO_3_ nanocavity at 25°C and 90°C with different cavity widths (w). The cavity length and thickness are fixed at 1300 and 40 nm, respectively. The incident light is fixed at 992 cm^−1^. The red wireframe corresponds to the near‐field imaging simulations at 25°C and 90°C in the experiments. (b) Optical distribution maps of simulated α‐MoO_3_ nanocavity at 25°C and 90°C with different cavity thicknesses (h). The cavity length and width are fixed at 1300 and 160 nm, respectively. The incident light is fixed at 992 cm^−1^. (c, d) The blue and red dots on the left side of the figure represent the extracted PhPs resonance wavelengths at 25°C and 90°C from (a), respectively. The blue and red dots on the right side of the figure represent the extracted PhPs resonance wavelengths at 25°C and 90°C from (b), respectively.

Subsequently, we fixed the width of the α‐MoO_3_ nanocavity at 160 nm, the length at 1302 nm, and the incident light wavenumber at 992 cm^−1^, and then conducted simulations by varying the thickness. As shown in Figure 5b,d, for VO_2_ at 25°C and 90°C, with the increase of cavity thickness, the resonance order decreases and the $\lambda_{p}$ of PhPs becomes longer. The simulation results show that the variation in cavity thickness can also tune the F‐P resonance mode of PhPs (more images are shown in Supporting Information S1: Figure S5). For cavity length, if it is excessively long, F‐P resonances cannot be sustained. Therefore, we selected a 900‐nm nanocavity and found that, under identical conditions, its resonance order is lower than that of the 1302‐nm nanocavity, as shown in Supporting Information S1: Figure S7. Simulations of a series of nanocavities with different widths and thicknesses, as well as optical imaging of nanocavities of different lengths, indicate that the cavity size has a tuning effect on the F‐P mode. Moreover, provided that the F‐P resonance condition is satisfied, wider, thinner, and longer nanocavities support higher‐order F‐P resonance. This geometric size tuning phenomenon can be attributed to the different electromagnetic confinement capabilities of α‐MoO_3_ of different sizes [44, 45, 46, 47, 48]. This provides a specific structural optimization direction for the design of high‐performance nanophotonic devices.

## 5 Conclusion

In summary, we have developed an intriguing method: by constructing α‐MoO_3_ nanocavity/VO_2_ heterojunctions, we realized the dynamically reversible modulation of Fabry–Pérot resonance of phonon polaritons in α‐MoO_3_ nanocavities via the dielectric property changes induced by the phase transition of VO_2_. Specifically, by using infrared nano‐imaging technology, we obtained the optical images of Fabry–Pérot resonance of phonon polaritons in α‐MoO_3_ nanocavities and identify their high sensitivity and precise tuning range. We analyzed the existence and state of phonon polaritons in α‐MoO_3_ nanocavities as well as their dispersion relations under near‐field imaging, the state of phonon polaritons before and after the phase transition of VO_2_, and the tuning effect of the nanocavity geometry on the Fabry–Pérot resonance of phonon polaritons. Our findings demonstrate that the phase transition of VO_2_ and the variation of α‐MoO_3_ nanocavity geometry exert controllable and reversible tuning effects on the Fabry–Pérot resonance modes of α‐MoO_3_ nanocavities; meanwhile, VO_2_ serves as a reliable platform for the resonance modulation of phonon polaritons in α‐MoO_3_ nanocavities. This achievement opens up a reliable new avenue for the tuning of Fabry–Pérot polaritonic resonators.

## 6 Methods

### 6.1 Experimental Section

A diluted colloid solution of α‐MoO_3_ nanocavities was evenly deposited onto the surface of a 50–nm VO_2_/Al_2_O_3_ heterojunction, and their optimal size was characterized via AFM. Near‐field optical properties were investigated using a commercial s‐SNOM, which is based on AFM technology. The experiment employed a Pt/Ir probe (Arrow‐NCPt‐50, Nano World) with a tip radius of approximately 20 nm. This probe operated in tapping mode, featuring a resonance frequency of around 255 kHz and a vertical oscillation amplitude of 100 nm. The illumination source was a tunable QCL. Its p‐polarized monochromatic beam was collimated using a parabolic mirror and focused onto the interaction zone between the probe and the sample. To minimize far‐field background interference, pseudo‐heterodyne interferometric phase detection was utilized to capture the light scattered by the probe. Background suppression was achieved through demodulation at the third harmonic of the tapping frequency.

### 6.2 Numerical Simulation

A systematic numerical investigation of the electromagnetic‐field distribution in an air/α‐MoO_3_ nanocavity/VO_2_ three‐layer structure was performed using the finite‐element method (FEM) implemented in COMSOL Multiphysics (Wave Optics Module). In the model, air constituted the top layer, the α‐MoO_3_ nanocavity served as the middle layer (with thickness and width treated as variables), and VO_2_ acted as the bottom layer. The optical response of α‐MoO_3_ was described by a Lorentz model, with parameters detailed in Supporting Information S1: Note S1, while the relative permittivities of VO_2_ in its insulating and metallic phases are shown in Supporting Information S1: Figure S6. The simulation geometry is illustrated in Supporting Information S1: Figure S8. The incident wavenumber was set to 992 cm^−1^ and could be varied as required. The out‐of‐plane (z‐direction) electric‐field intensity was extracted at a height of 50 nm above the α‐MoO_3_ nanocavity. In addition, the dispersion relations of the α‐MoO_3_ nanocavity along the [001] direction, before and after the VO_2_ phase transition, were calculated by solving the complex refractive index of the air/α‐MoO_3_ nanocavity/VO_2_ system using the Fresnel formalism, as described in Supporting Information S1: Note S2. These numerical results provide quantitative insights into the mechanism by which the VO_2_ phase transition modulates the F‐P cavity.

## Author Contributions

Jianing Chen supervised the work. Yongqian Zhao and Jianing Chen conceived the study. Chen Ge provided the 50 nm‐thick VO_2_ film. Zhiqiang Shi and Yongqian Zhao collaboratively conducted near‐field optical imaging, SEM characterization, polarized Raman spectra, and performed data analysis and validation. Ying Liao and Rao Fu constructed the numerical simulation models. Yongqian Zhao, Zhiqiang Shi, Shuai Feng and Jianing Chen wrote the original manuscript, and all authors discussed the results and provided comments on the manuscript.

## Conflicts of Interest

The authors declare no conflicts of interest.

## Supporting information


Supporting Information S1

## Data Availability

Research data are not shared.
