# Supplementary material for: Reversible and Dynamic Tuning Mechanism for Fabry–Pérot Polaritonic Resonators
Source: Nanophotonics. 2026 Apr 16;15(8):e70074. doi: 10.1002/nap2.70074 (PMC13642106; doi:10.1002/nap2.70074)
Supplement: Supplementary file 1 [file NAP2-15-e70074-s001.docx]

**Supporting Information for:**

Reversible and dynamic tuning mechanism for Fabry-Pérot polaritonic resonators

Zhiqiang Shi^1^, Yongqian Zhao^2,3,^*, Ying Liao^5^, Rao Fu^2,3^, Chen Ge^2,3^, Shuai Feng^1,^*, and Jianing Chen^2,3,4,^*

^1^School of Science, Minzu University of China, Beijing 100081, China

^2^Beijing National Laboratory for Condensed Matter Physics, Institute of Physics, Chinese Academy of Sciences, Beijing 100190, China

^3^School of Physical Sciences, University of Chinese Academy of Sciences, Beijing 100049, China

^4^Songshan Lake Materials Laboratory, Dongguan 523808, China

^5^Suzhou Laboratory, Suzhou, Jiangsu 215123, China.

^*^Corresponding author: Yongqian Zhao (yqzhao@iphy.ac.cn); Shuai Feng (fengshuai75@163.com); Jianing Chen (jnchen@iphy.ac.cn)

**Table of Contents**

**Note S1.** Calculation of the relative permittivity of α-MoO_3_

**Note S2.** The dispersion relations of the PhPs along the [001] crystal direction of α-MoO_3_ on the VO_2_ film at 25 ℃ and 90 ℃

**Figure S1.** Tuning of the F-P mode within the range of 989.5 cm^-1^ to 998.5 cm^-1^ for the incident light

**Figure S2.** The extraction of the effective reflection phase of α-MoO_3_ PhPs, when VO_2_ is at 25 ℃ and 90 ℃, respectively

**Figure S3.** Temperature-tuned F-P mode (a complete cycle)

**Figure S4.** A complete series of simulated optical images of α-MoO_3_ nanocavities with a length of 1300 nm, a thickness of 40 nm, and varying widths *w*

**Figure S5.** A complete series of simulated optical images of α-MoO_3_ nanocavities with a length of 1300 nm, a width of 160 nm, and varying thicknesses *h*

**Figure S6.** The optical properties of the insulating phase and metallic phase VO_2_

**Figure S7.** Near-field optical imaging and morphology of 900 nm-long nanocavity in the range of 994–1000 cm^-1^

**Figure S8.** Simplified illustration of the modeling of the three-layer structure of air/α-MoO_3_ nanocavity/VO_2_

**References**

**Note S1. Calculation of the relative permittivity of α-MoO_3_.**

The anisotropic dielectric constant of α-MoO_3_ is discussed here. In the mid-infrared region, the optical response of α-MoO_3_ is dominated by phonon absorption rather than electronic transitions[1-4], and its dielectric function can be described by the following Lorentz equation:

$$\begin{aligned} \varepsilon_{j}=\varepsilon_{\infty}^{j}\left( 1+\frac{{\omega_{LO}^{j}}^{2}-{\omega_{TO}^{j}}^{2}}{{\omega_{TO}^{j}}^{2}-\omega^{2}-i\omega\Gamma^{j}} \right),j=a,b,c \end{aligned}$$

Here, $\varepsilon_{j}$ denotes the principal components of the dielectric tensor, $\varepsilon_{\infty}^{j}$ represents the high-frequency dielectric constant, $\omega_{LO}^{j}$ corresponds to the longitudinal optical phonon frequency, $\omega_{TO}^{j}$ indicates the transverse optical phonon frequency, *ω* signifies the frequency of the incident light, and $\Gamma^{j}$ refers to the damping factor. Additionally, *a*, *b*, and *c* are associated with the three crystallographic directions [100], [001], and [010] of α-MoO_3_, respectively.

**Parameters required for calculating the relative permittivity (unit: cm^-1^) :**

| Direction | [100]  Ref. [1] | [100]  this work | [001]  Ref. [1] | [001]  this work | [010]  Ref. [1] | [010]  this work |
| --- | --- | --- | --- | --- | --- | --- |
| $\boldsymbol{\varepsilon}_{\boldsymbol{\infty}}$ | 4.0 | 5.0 | 5.2 | 5.8 | 2.4 | 3 |
| $\boldsymbol{\omega}_{\boldsymbol{LO}}$ | 972 | 980 | 851 | 851 | 1004 | 1004 |
| $\boldsymbol{\omega}_{\boldsymbol{TO}}$ | 820 | 818 | 545 | 545 | 958 | 960 |
| $\boldsymbol{\Gamma}$ | 4 | 4 | 4 | 4 | 2 | 1.5 |

**Note S2. The dispersion relations of the PhPs along the [001] crystal direction of α-MoO_3_ on the VO_2_ film at 25 ℃ and 90 ℃.**

To analyze the dispersion of PhPs in α-MoO_3_ nanocavity, we calculated the complex reflectivity $r_{p}(\omega,q)$ of the air/α-MoO_3_ nanocavity/ VO_2_ three-layer structure[4]. The total reflectivity of this system can be expressed as:

$$r_{p}=\frac{r_{a}+r_{s}e^{i2k_{2}^{b}d}}{1+r_{a}r_{s}e^{i2k_{2}^{b}d}}$$

$$r_{a}=\frac{\varepsilon_{y}k_{a}^{b}-\varepsilon_{a}k_{2}^{b}}{\varepsilon_{y}k_{a}^{b}+\varepsilon_{a}k_{2}^{b}}$$

$$r_{s}=\frac{\varepsilon_{s}k_{2}^{b}-\varepsilon_{y}k_{s}^{b}}{\varepsilon_{s}k_{2}^{b}+\varepsilon_{y}k_{s}^{b}}$$

In the equation, the subscripts “*a*”, “2”, and “*s*” respectively represent air, α-MoO_3_, and insulating VO_2_. $r_{a}$ and $r_{s}$ respectively represent the reflectivity of the air/α-MoO_3_ interface and the α-MoO_3_/ VO_2_ interface. $\varepsilon_{a}$, $\varepsilon_{s}$ and $\varepsilon_{y}$ respectively represent the dielectric constants of air, insulating VO_2_ and α-MoO_3_ parallel to the [001] crystal direction. The $k_{i}^{b}$ (*i* = *a*, 2, *s*) indicates the momentum along the b-axis in the *i*-th layer. When *i* = 2, the corresponding momentum is $k_{2}^{b}=\sqrt{\varepsilon_{y}\frac{\omega^{2}}{c^{2}}-\frac{\varepsilon_{y}}{\varepsilon_{z}}q^{2}}$; for *i* = *a* or *s*, the momentum is $k_{i}^{b}=\sqrt{\varepsilon_{i}\frac{\omega^{2}}{c^{2}}-q^{2}}$. The *d* represents the thickness of the α-MoO_3_ nanocavity.

**Figure S1. Tuning of the F-P mode within the range of 989.5 cm^-1^ to 998.5 cm^-1^ for the incident light.** (a, b) Near-field optical images of a single α-MoO_3_ nanocavity at 25 ℃ (resonance order 10 to 2) and 90 ℃, respectively. (c, d) Line profiles along the axis of the nanocavity corresponding to (a) and (b) images, respectively.

**Figure S2. The extraction of the effective reflection phase of α-MoO_3_ PhPs, when VO_2_ is at 25 ℃ and 90 ℃, respectively.** (a, b) The small blue and red circles, respectively, represent the $(2L/\lambda_{p})$**-***n* relationship extracted from Figure S1a,b. The blue and red dotted lines are the fitting lines, and their y-axis intercept is${\Delta\varphi}/\pi$, the value of $\Delta\varphi$ is approximately equal to *π*.

**Figure S3.** **Temperature-tuned F-P mode (a complete cycle).** (a, b) Optical images of the α-MoO_3_ nanocavity/ VO_2_ at 992 cm^-1^ during complete gradient heating (a) and cooling (b). Scale bar: 300 nm. (c, d) Line profiles extracted from (a) and (b), respectively.

**Figure S4.** **A complete series of simulated optical images of α-MoO_3_ nanocavities with a length of 1300 nm, a thickness of 40 nm, and varying widths *w.*** (a) Optical imaging simulation of α-MoO_3_ nanocavities on VO_2_ at 25 ℃, with varying widths under a fixed incident wavenumber at 992 cm^-1^. (b) Optical imaging simulation of α-MoO_3_ nanocavities on VO_2_ at 90 ℃, with varying widths under a fixed incident wavenumber at 992 cm^-1^.

**Figure S5.** **A complete series of simulated optical images of α-MoO_3_ nanocavities with a length of 1300 nm, a width of 160 nm, and varying thicknesses *h*.** (a) Optical imaging simulation of α-MoO_3_ nanocavities on VO_2_ at 25 ℃, with only the thickness varying and the $\omega$ fixed at 992 cm^-1^. (b) Optical imaging simulation of α-MoO_3_ nanocavities on 90 ℃ VO_2_, with only the thickness varied and the $\omega$ fixed at 992 cm^-1^.

**Figure S6.** **Optical parameters of VO_2_ substrates in the insulating and metallic phases, and the PhPs dispersion along the [001] direction of α-MoO_3_ supported on them** [5]**.** (a) The real parts of the relative permittivity of the insulating phase and the metallic phase of VO_2_ in the 8.5–11.5 $\mu$m band, respectively. (b) The imaginary parts of the relative permittivity of the insulating phase and the metallic phase of VO_2_ in the 8.5–11.5 $\mu$m band. (c) The dispersion relations of the α-MoO_3_ nanocavity PhPs on the insulating and metallic phases of VO_2_ in the range of 960 to 1020 cm^-1^.

**Figure S7.** **Near-field optical imaging and morphology of 900 nm-long nanocavity in the range of 994–1000 cm^-1^.** (a) Near-field optical imaging of a 900 nm nanocavity at room temperature in the range of 994–1000 cm^-1^. (b) Near-field optical imaging of a 900 nm nanocavity at 90 °C in the range of 994–1000 cm^-1^. (c) AFM image of the nanocavity. (d) The contour lines of the nanocavity extracted from Figure c.

This indicates that the F-P resonance of nanocavities of different lengths is also modulated by the VO_2_ phase transition. The F-P resonance order of the 1302 nm nanocavity at 25 ℃ and 994 cm^-1^ is 6, while that of the 900 nm nanocavity under the same conditions is 5, indicating that the length can regulate the F-P resonance order of the nanocavity.

**Figure S8.** **Simplified illustration of the modeling of the three-layer structure of air/α-MoO_3_ nanocavity/VO_2_.** The upper part is an air medium, the middle layer is an α-MoO_3_ nanocavity (whose thickness and width are set as variables), with a relative permittivity determined by the Lorentz model; the bottom is the phase change material VO_2_, whose relative permittivities in the insulating and metallic states are shown in Figure S6.

**References**

1.	Z. B. Zheng, N. Xu, S. L. Oscurato, et al., “A mid-infrared biaxial hyperbolic van der Waals crystal,” *Science Advances* 5, no. 5 (2019): eaav8690, https://doi.org/doi:10.1126/sciadv.aav8690.

2.	W. L. Ma, P. Alonso-Gonzalez, S. Li, et al., “In-plane anisotropic and ultra-low-loss polaritons in a natural van der Waals crystal,” *Nature* 7728, no. 562 (2018): 557-652, https://doi.org/10.1038/s41586-018-0618-9.

3.	H. Hu, N. Chen, H. C. Teng, et al., “Gate-tunable negative refraction of mid-infrared polaritons,” *Science* 6632, no. 379 (2023): 558-561, https://doi.org/10.1126/science.adf1251.

4.	Y. Q. Zhao, G. Li, Y. Y. Yao, et al., “Tunable heterostructural prism for planar polaritonic switch,” *Science Bulletin* 16, no. 68 (2023): 1757-1763, https://doi.org/10.1016/j.scib.2023.07.024.

5.	C. Wan, Z. Zhang, D. Woolf, et al., “On the Optical Properties of Thin-Film Vanadium Dioxide from the Visible to the Far Infrared,” *Annalen Der Physik* 10, no. 531 (2019): 1900188, https://doi.org/10.1002/andp.201900188.
